# A case of ramucirumab-related gastrointestinal perforation in gastric cancer with small bowel metastasis

**DOI:** 10.1186/s40792-017-0399-7

**Published:** 2017-12-19

**Authors:** Shinya Urakawa, Daisuke Sakai, Yasuhiro Miyazaki, Toshihiro Kudo, Aya Katou, Chiaki Inagaki, Koji Tanaka, Tomoki Makino, Tsuyoshi Takahashi, Yukinori Kurokawa, Makoto Yamasaki, Kiyokazu Nakajima, Shuji Takiguchi, Taroh Satoh, Masaki Mori, Yuichiro Doki

**Affiliations:** 1 Department of Gastroenterological Surgery, Graduate School of Medicine, Osaka University, 2-2, E-2, Yamadaoka, Suita, Osaka 565-0871 Japan; 2 Department of Frontier Science for Cancer and Chemotherapy, Osaka University Graduate School of Medicine, E21-19, 2-2, Yamadaoka, Suita, Osaka Japan; 3 Division of Next Generation Endoscopic Intervention (Project ENGINE), Global Center for Medical Engineering and Informatics, Center of Medical Innovation and Translational Research, Osaka University, 2-2, Yamadaoka, Suita, Osaka 565-0871 Japan

**Keywords:** Ramucirumab, Gastrointestinal perforation, Gastric cancer, Small bowel metastasis

## Abstract

**Background:**

Ramucirumab is a monoclonal antibody targeting vascular endothelial growth factor receptor 2 (VEGFR-2). Ramucirumab either alone or in combination with paclitaxel (PTX) has been found to be safe and effective for patients with previously treated advanced gastric cancer. One of the serious adverse events associated with ramucirumab is gastrointestinal (GI) perforation.

**Case presentation:**

We report the case of a 67-year-old man who developed a ramucirumab-related GI perforation while undergoing treatment for gastric cancer with small bowel metastasis. He underwent laparoscopic total gastrectomy following neoadjuvant chemotherapy in January 2015 and was diagnosed with hepatic and bone recurrence in October 2015. Ramucirumab in combination with PTX was administered for one and half months after first-line chemotherapy failure. He presented with abdominal pain 7 days after the last ramucirumab dose, and emergency exploratory surgery revealed a small intestinal perforation. Pathological findings indicated that it occurred in a zone containing a small intestinal tumor, which was found to be metastasis of the gastric cancer. He had no postoperative complications, but chemotherapy was not reintroduced and he died 3 months later.

**Conclusion:**

We present a recent case of ramucirumab-related gastrointestinal perforation in gastric cancer with small bowel metastasis. This case is rare, but important to consider.

## Background

Ramucirumab (Cyramza; IMC-1121B; LY3009806; Lilly Oncology, Lilly Research & Development, New York, NY, USA) is a monoclonal antibody targeting vascular endothelial growth factor receptor 2 (VEGFR-2) [1]. VEGFR-2 inhibition reduces tumor vascularity and growth in animal models [2]. Ramucirumab alone or in combination with paclitaxel (PTX) was proven safe and effective for administration after first-line chemotherapy of advanced gastric cancer and gastroesophageal junction adenocarcinoma [3, 4]. Ramucirumab is known to be associated with hypertension, proteinuria, bleeding, and gastrointestinal (GI) perforation [3–7], a rare but life-threatening and emergent adverse event [8].

Here, we report a case of ramucirumab-related gastrointestinal perforation in gastric cancer with small bowel metastasis.

## Case presentation

In November 2014, a 67-year-old man was referred to our hospital due to gastric cancer with concurrent left adrenal metastasis. PET scan revealed high FDG uptake at the primary tumor (maximum standardized uptake value (SUV-max), 10.2) and at the left adrenal gland (SUV-max, 4.5). He had no prior or family history of cancer. Following combination chemotherapy with docetaxel, cisplatin, and S-1, he achieved partial response; moreover, PET-CT showed regression of FDG uptake at the primary tumor (SUV-max, 7.5) and disappeared at the left adrenal. No other metastasis was detected. Secondary laparoscopy revealed no peritoneal dissemination and negative with peritoneal washing cytology. Therefore, he underwent laparoscopic total gastrectomy with D2 lymph node dissection (Roux-en-Y reconstruction) and combined resection of the left adrenal gland in January 2015. The pathological diagnosis was moderately differentiated adenocarcinoma, 40 × 30 mm, ypT4a(SE), ly2, v0, ypN0(0/19), ypM1(ADR), Grade 1a, and HER2-positive.

**Fig. 1:**
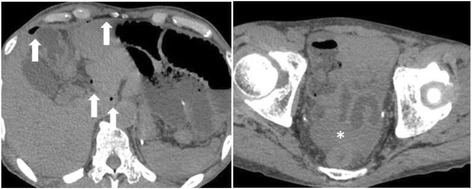
Free air and fluid in the peritoneal cavity visible on abdominal CT

**Fig. 2:**
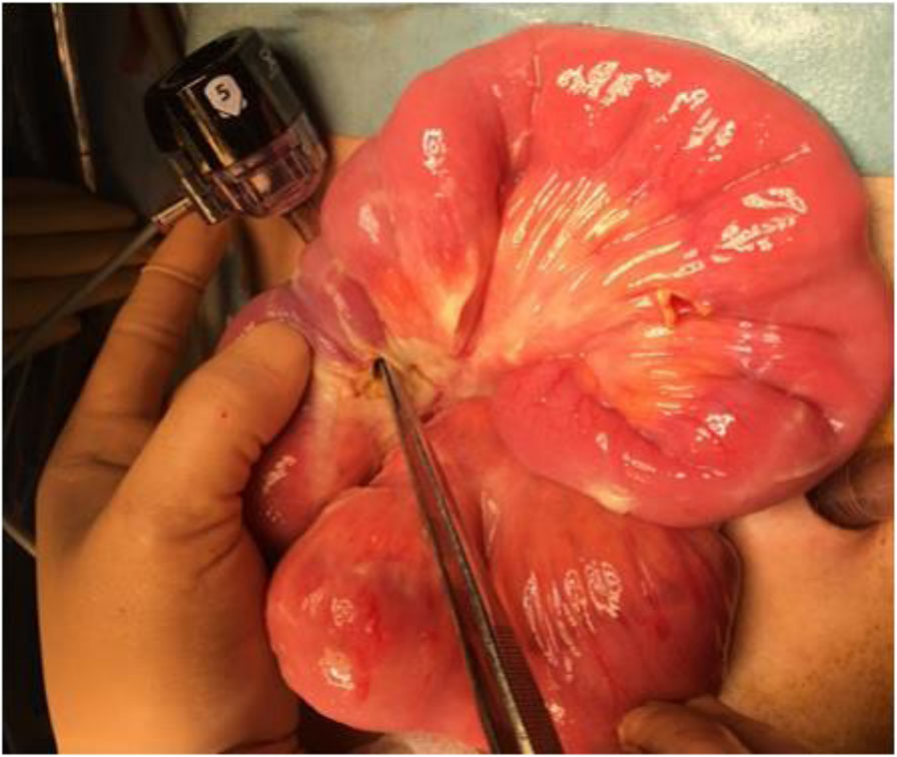
Intraoperative findings revealed a large amount of dirty fluid in the abdominal cavity. A small bowel perforation was identified 50 cm distal from the site of jejuno-jejunal anastomosis(Roux-en-Y reconstruction)

He was diagnosed with hepatic and bone recurrence during S-1 adjuvant chemotherapy in October 2015 and underwent first-line chemotherapy with cisplatin, S-1, and trastuzumab. After first-line chemotherapy failure, he received two courses of ramucirumab at 8 mg/kg and PTX at 80 mg/m^2^ beginning on April 12, 2016. His PS was 0 at the beginning of second-line chemotherapy. May 31, 2016, on the appointment date, 1 week after the last ramucirumab administration, he visited to our hospital with abdominal pain which onset was 3 days ago. A physical examination revealed left lower quadrant pain and rebound tenderness. He had a blood pressure of 125/75 mmHg, body temperature of 38.2 °C, a white blood count of 4400/mm^3^, and a C-reactive protein level of 33.63. An abdominal CT-scan showed free air and fluid, but no intestinal obstruction (Fig. 1). Emergency exploratory surgery was performed under the impression of peritonitis, and the surgery revealed a large amount of dirty fluid throughout the abdominal cavity. A small intestinal perforation was identified 50 cm distal from the site of jejuno-jejunal anastomosis (Roux-en-Y reconstruction) (Fig. 2), and there was no peritoneal dissemination. We performed small bowel segmental resection and functional end-to-end anastomosis. He had no postoperative complications and was transferred to another hospital on post-operative day 23 for radiation for his bone metastasis. He was introduced to palliative care for disease progression following the radiation treatment and died 3 months later.

**Fig. 3:**
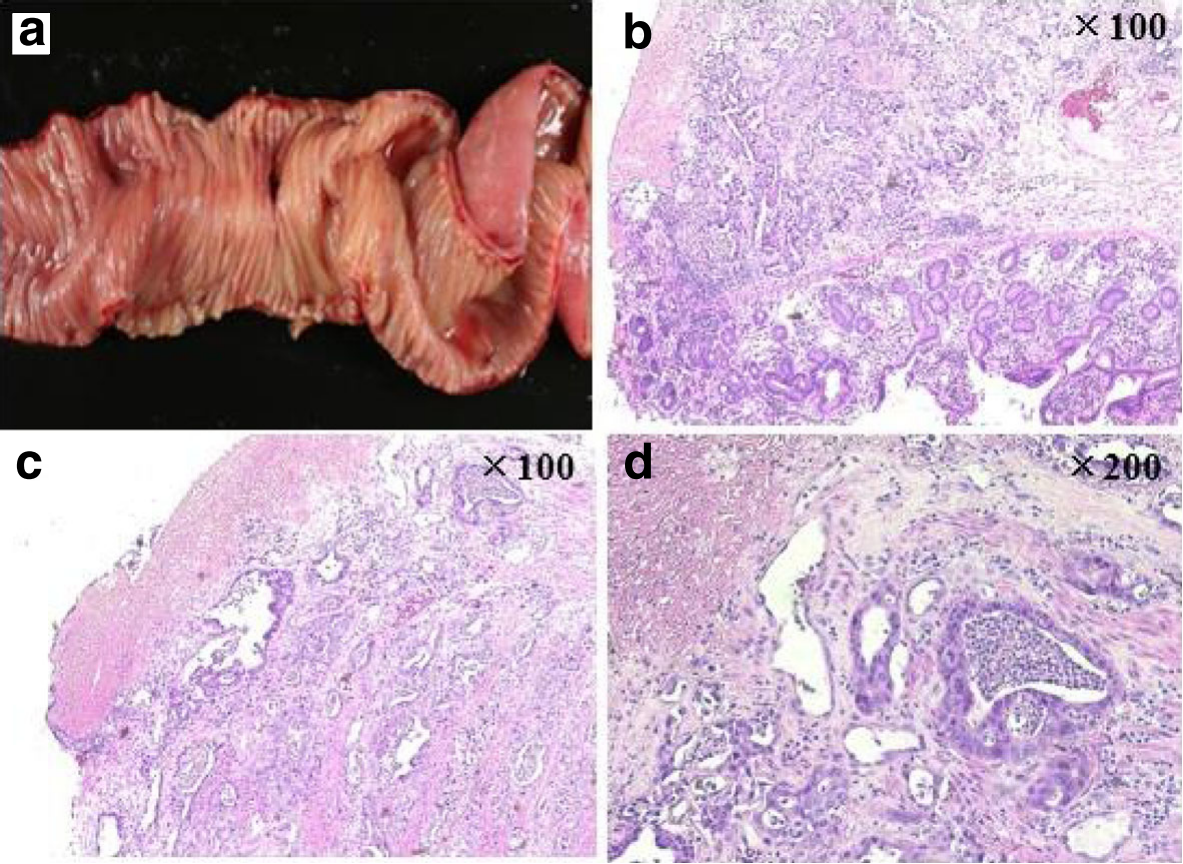
**a**–**d** Macroscopic and microscopic findings showed that GI perforation occurred in an area of tumor tissue and that invasive adenocarcinoma was found from the submucosa to the subserosa, rather than in the epithelial cells

The macroscopic findings indicated that the perforation occurred in the resected small bowel segment. No neoplastic lesions were found, but the microscopic findings showed that invasive adenocarcinoma had infiltrated from the submucosa to the subserosa, rather than the epithelial cells. Degeneration and necrosis due to chemotherapy were found in the tumor tissue (Fig. 3).

### Discussion

Gastric cancer is the fourth most common malignant disease and the second leading cause of cancer mortality worldwide [9]. The first-line chemotherapies for unresectable advanced gastric cancer or recurrent gastric cancer are fluoropyrimidine-based and platinum-based combinations [10]. Ramucirumab is a human IgG1 monoclonal antibody VEGFR-2 antagonist [1] and was proven to be effective for advanced gastric cancer. Ramucirumab alone or in combination with PTX prolonged survival in two randomized controlled trials including patients with previously treated advanced stomach cancer and gastroesophageal junction adenocarcinoma (REGARD and RAINBOW) [3, 4]. Ramucirumab is generally well tolerated and has acceptable toxicity that includes hypertension, proteinuria, and minor hemorrhage [3–7]. GI perforation is a rare but serious adverse event. The incidence of all-grade GI perforation with ramucirumab was 1.5% (95% CI 1.1–2.1%) with a mortality of 29.8% (95% CI 14.9–50.7%) in a previous study [8].

However, the risk factors for GI perforation with ramucirumab have not yet been investigated, and bevacizumab, an anti-angiogenesis inhibitor-like ramucirumab, is also known to increase the risk of GI perforation. Several clinical factors, including peptic ulcer disease, diverticulitis, intestinal obstruction, underlying cancer, recent endoscopy, abdominal radiotherapy, steroid or nonsteroidal anti-inflammatory drug (NSAID) use, and bowel resection at surgery, were considered to increase the risk of GI perforation in patients treated with bevacizumab [11–15]. These risk factors could lead to a recommendation for a different antiangiogenic agent treatment, such as ramucirumab. In the case presented here, the GI perforation was related to a small bowel metastasis.

Gastric cancer metastasis to the small intestine is very rare, and almost all metastases to the small intestine are direct invasions or disseminations, making lymphatic or hematogenous metastases especially rare (9.1% of metastatic tumors to the small intestine) [16]. In this case, the tumor in the small intestine was considered a hematogenous metastasis of the gastric cancer because (1) this tumor was histologically the same as the gastric cancer, (2) there was no peritoneal dissemination, and (3) hematogenous spread is more typical and often includes the adrenal gland, liver, and bone.

The mechanisms of ramucirumab-related GI perforation remain unclear, and the current understanding is based primarily on other anti-angiogenesis inhibitors. One mechanistic hypothesis is tumor necrosis, which would lead to a weakening of the intestinal wall [17]. This hypothesis could account for the perforation reported here, since it occurred in an area of neoplastic infiltration of the small intestine. A second hypothesis is intestinal mesenteric vessel thrombosis or vasoconstriction, which could cause bowel ischemia [18, 19]. We observed these symptoms in macro- and microscopic findings in our case.

Ramucirumab-related GI perforation is difficult to manage since both antiangiogenic agent treatment and the chemotherapy-related immunocompromised condition of the patient impair wound healing. The choice of a conservative or operative approach should be based on the condition of the individual [11], and whether ramucirumab should be reintroduced is uncertain.

It is difficult to detect an intestinal lesion as small as the one presented here, but the best approach to ramucirumab-related gastrointestinal perforation remains surveillance and the prevention of clinical risk factors.

## Conclusions

We present a recent case of ramucirumab-related gastrointestinal perforation in gastric cancer with small bowel metastasis. This case is rare, but important to consider.

## Abbreviations

GI: Gastrointestinal
PTX: Paclitaxel
VEGFR-2: Vascular endothelial growth factor receptor 2
